# Unrecognised actionability for breast cancer risk variants identified in a national-level review of Australian familial cancer centres

**DOI:** 10.1038/s41431-024-01705-9

**Published:** 2024-10-14

**Authors:** Cristina Fortuno, Elisa J. Cops, Aimee L. Davidson, Johanna Hadler, Giovanni Innella, Maddison E. McKenzie, Michael Parsons, Ainsley M. Campbell, Andrew Dubowsky, Verna Fargas, Michael J. Field, Helen G. Mar Fan, Cassandra B. Nichols, Nicola K. Poplawski, Linda Warwick, Rachel Williams, Victoria Beshay, Caitlin Edwards, Andrea Johns, Mary McPhillips, Vanessa Siva Kumar, Rodney Scott, Mark Williams, Hamish Scott, Paul A. James, Amanda B. Spurdle

**Affiliations:** 1 Population Health Program, QIMR Berghofer Medical Research Institute, Brisbane, QLD Australia; 2 Parkville Familial Cancer Centre, Peter MacCallum Cancer Centre and Royal Melbourne Hospital, Melbourne, VIC Australia; 3 Dipartimento di Scienze Mediche e Chirurgiche, Università di Bologna, Bologna, Italy; 4 Department of Clinical Genetics, Austin Health, Melbourne, VIC Australia; 5 Department of Genetics and Molecular Pathology, SA Pathology, Adelaide, SA Australia; 6 College of Medicine and Public Health, Flinders University, Adelaide, SA Australia; 7 Liverpool Cancer Genetics, Liverpool Hospital, Liverpool, NSW Australia; 8 Family Cancer Clinic, Royal North Shore Hospital, St Leonards, NSW Australia; 9 Genetic Health Queensland Royal Brisbane and Women’s Hospital, Brisbane, QLD Australia; 10 Faculty of Medicine, The University of Queensland, Brisbane, QLD Australia; 11 Genetic Health WA, King Edward Memorial Hospital, Perth, WA Australia; 12 Adult Genetics Unit, Royal Adelaide Hospital, Adelaide, SA Australia; 13 Adelaide Medical School, University of Adelaide, Adelaide, SA Australia; 14 ACT Genetic Service Canberra Health Services, Garran, ACT Australia; 15 School of Clinical Medicine, UNSW Medicine & Health, UNSW Sydney, Sydney, NSW Australia; 16 Prince of Wales Hereditary Cancer Centre, Prince of Wales Hospital, Randwick, NSW Australia; 17 Peter MacCallum Cancer Centre, Melbourne, VIC Australia; 18 Diagnostic Genomics, Pathwest Laboratory Medicine, QEII Medical Centre, Nedlands, WA Australia; 19 NSW Health Pathology, Newcastle, NSW Australia; 20 Invitae Australia, Sydney, NSW Australia; 21 Hunter Medical Research Institute, University of Newcastle, Newcastle, NSW Australia; 22 Genomic Diagnostics, Melbourne, VIC Australia; 23 Centre for Cancer Biology, An alliance between SA Pathology and the University of South Australia, Adelaide, SA Australia; 24 UniSA Clinical and Health Sciences, University of South Australia, Adelaide, SA Australia; 25 Sir Peter MacCallum Department of Oncology, University of Melbourne, Melbourne, VIC Australia; 26 Faculty of Medicine, The University of Queensland, Herston, QLD Australia

**Keywords:** Cancer genetics, Genetic testing

## Abstract

Breast cancer remains a significant global health challenge. In Australia, the adoption of publicly-funded multigene panel testing for eligible cancer patients has increased accessibility to personalised care, yet has also highlighted the increasing prevalence of variants of uncertain significance (VUS), complicating clinical decision-making. This project aimed to explore the spectrum and actionability of breast cancer VUS in Australian familial cancer centers (FCCs). Leveraging data from 11 FCCs participating in the Inherited Cancer Connect database, we retrieved VUS results from 1472 patients. Through ClinVar crosschecks and application of gene-specific ACMG/AMP guidelines, we showed the potential for reclassification of 4% of unique VUS as pathogenic or likely pathogenic, and 80% as benign or likely benign. Surveys conducted with FCCs and diagnostic laboratories described current practices and challenges in variant reclassifications, highlighting resource constraints preventing periodic VUS review and notifications from the laboratories to the FCCs. Our study suggests there are benefits to routine VUS review and reclassification, particularly in publicly-funded healthcare systems. Future research should focus on assessing the clinical impact and cost-effectiveness of implementing routine variant review practices, alongside efforts to enhance communication between FCCs and laboratories.

## Introduction

Breast cancer is the most prevalent cancer diagnosed in women worldwide. The proportion of breast cancers in the general population attributed to *BRCA1* and *BRCA2* germline pathogenic variants is approximately 5–10% [1, 2], but this proportion is much higher (up to 55%) in selected families referred to familial cancer clinics [3]. Even though rarer than *BRCA1* and *BRCA2*, a short list of other highly penetrant genes are known to elevate breast cancer risk [4]. In general, hereditary breast cancer is associated with poorer survival rates compared to breast cancer as a whole [5, 6].

Genetic testing is valuable as it provides patients and their close relatives with knowledge about their hereditary cancer risk, and guides personalised strategies for cancer prevention and treatment for both pathogenic variant carriers and those with “normal” results (no germline pathogenic variant identified). Multigene panel testing has been adopted as a cost-effective strategy to simultaneously sequence multiple genes; however, these panels reveal variants of uncertain significance (VUS) more frequently than single gene testing approaches. Identification of VUS presents an important challenge to the clinical utility of precision genomic medicine [7]. These variants not only limit effective clinical management but are also a source of anxiety and confusion for some clinicians, and for patients and their family members [8]. Presently, there are no definitive recommendations regarding the reporting of VUS, and attitudes on their disclosure vary among clinical and laboratory genetic counsellors [9].

Testing of breast cancer predisposition genes increased significantly in Australia following the introduction of national government funding of multigene panel testing in 2017. The uptake of multigene panel testing is expected to have led to a corresponding increase in the number of breast cancer gene VUS identified [10]. ClinVar is a comprehensive resource that aggregates variant data from multiple sources, including clinical laboratories, and thus provides a useful overview of the diversity and classification of variants encountered in clinical and research settings [11]. For the subset of genes whose testing is publicly funded in Australia (*BRCA1, BRCA2, CDH1, PALB2, PTEN, TP53*), the proportion of unique variants in ClinVar with aggregate clinical interpretation as VUS ranges from 16% to (*BRCA1*) to 47% (*CDH1*) (ClinVar, accessed 21 April 2024). Furthermore, when variants with conflicting classifications are taken into account, these two categories collectively constitute at least a third of all the variants submitted to ClinVar for each gene.

VUS reclassification has potential to alter clinical management in hereditary cancer patients [12]; however, little is known about the prevalence and potential for VUS reclassifications among breast cancer patients. The rate of VUS reclassification from purposeful variant reclassification efforts using American College of Medical Genetics and Genomics/Association for Molecular Pathology (ACMG/AMP) guidelines was 61% for a study of 92 patients with any VUS received through clinical testing [13], with ranges of up to 81.3% for *BRCA1/2*-focused studies [14–18]. It is therefore reasonable to think that reclassification of VUS in breast cancer risk genes has potential to inform patient clinical management.

The aim of this project was to identify the scope of the problem of breast cancer VUS in Australian familial cancer centres (FCCs), and to understand reclassification processes and interaction between FCCs and diagnostic laboratories, in order to consider future strategies for increased actionability and improved clinical management of breast cancer patients.

## Materials and methods

**Fig. 1:**
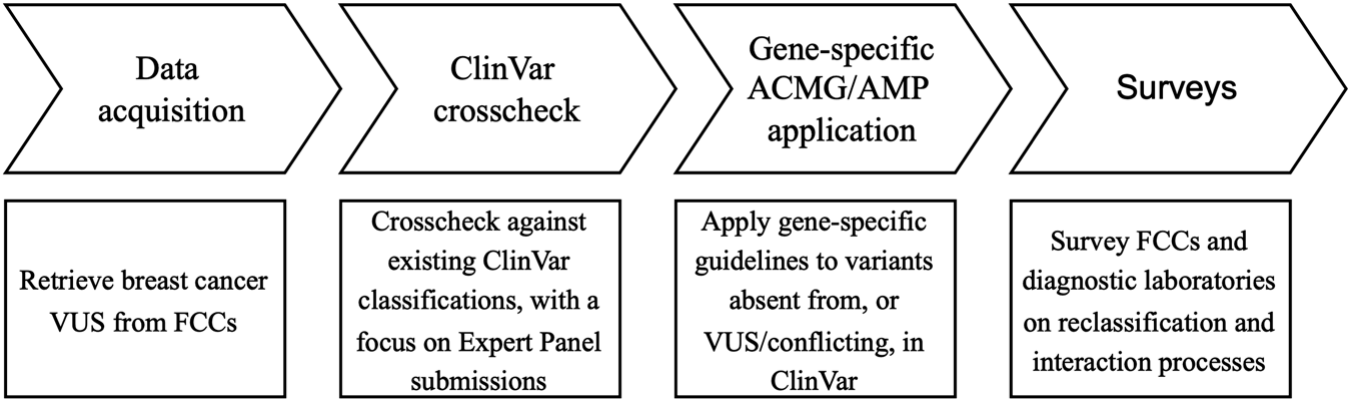
Overview of the process followed in this study to retrieve and review breast cancer VUS identified from FCC clinical databases for unrecognised actionability, and to understand processes limiting VUS review and reclassification. ACMG/AMP American College of Medical Genetics and Genomics/Association for Molecular Pathology, FCC Familial cancer centre, VUS Variant of uncertain significance.

An overview of the study process is shown in Fig. 1.

### Data acquisition

We engaged with 11 Australian FCCs participating in the Inherited Cancer Connect (ICCon) partnership (Forrest, 2018) to retrieve the breast cancer gene variants listed as VUS in their internal databases in the following genes eligible for publicly-funded testing in Australia: *BRCA1, BRCA2, CDH1, PALB2, PTEN*, and *TP53*. The participating FCCs were: ACT Genetic Service, Austin Health, the Adult Genetics Unit (a state-wide service for South Australia), Genetic Health Queensland, Genetic Health WA, Liverpool Cancer Genetics, Peter MacCallum Cancer Centre, Princes of Wales Hospital, Royal Melbourne Hospital, Royal North Shore, and St. George Hospital. Data retrieved corresponded to VUS results for patients undergoing multigene panel testing due to hereditary cancer risk identified post-2017 and up to 2022.

For the six selected genes, we received VUS results from 1516 patients. Patient data records, stored in the clinical databases of the FCCs using proprietary software (such as Progeny, TrakGene, FamBIS) were internally queried by FCC data managers using a database search function for terms to identify variants for any patient annotated with classification interpreted to be VUS in one of the genes of interest; the specific terms were selected according to the data dictionary for the relevant FCC, e.g., Class 3, VUS, VOUS. We first checked for nomenclature errors using the Mutalyzer tool [19]. Entries for 44 patients were unresolvable and were excluded, leaving a total of 1472 patients available for analyses. The nomenclature for the remaining entries was standardized as per HGVS recommendations.

### ClinVar crosscheck and groupings

To ascertain the extent to which variants labelled as “VUS” in FCC internal databases may have resulted from outdated or inadequately reassessed historical reports, we initially examined their classifications in ClinVar (as at September 2022). These variants were categorised into three distinct groups, as follows:

- Variants with Expert Panel submissions as Likely pathogenic and Pathogenic (hereafter referred to as P/LP) or Likely benign and Benign (hereafter referred to as B/LB)
- Variants with multiple non-conflicting submissions as P/LP or B/LB
- Variants not belonging to any of these groups (i.e. variants with a single submission as P/LP or B/LB, variants with conflicting submissions, variants classified as Uncertain, and variants not previously submitted to ClinVar), hereafter referred to as “Other”

### Gene-specific ACMG/AMP guidelines application

We applied gene-specific ACMG/AMP criteria to variants in the “Other” ClinVar group, using the following criteria specifications versions: BRCA1 v1.1.0, BRCA2 v1.1.0, CDH1 v3.1.0, PALB2 v1.1.0, PTEN v3.0.0, and TP53 v2.0.0. Details for specifications are available in the ClinGen Criteria Specification Registry (https://cspec.genome.network/cspec/ui/svi/). For the population-based codes (BA1, BS1, PM2), we used the gnomAD v4.1 database [20], except for BRCA1 and BRCA2 for which we performed this step prior to v4 release, and whose latest guidelines specify the use of gnomAD v2.1 and gnomAD v3.1 (non-cancer datasets). For all genes, the cutoffs used for SpliceAI splicing predictions were ≥0.2 (predicted impact) and ≤0.1 (no predicted impact), as recommended by the ClinGen Sequence Variant Interpretation Splicing Subgroup [21]. All other codes were assigned following the respective gene specifications. The proposed class following review was assigned based on a Bayesian point systems [22], which included reclassifying as LB variants with a total of −1 points where the only pathogenic code applied is PM2_Supporting, and there are at least two benign codes applied.

### Surveys to FCCs and diagnostic laboratories

To enhance comprehension of the VUS review process and the dynamics between FCCs and diagnostic laboratories in Australia, as well as to identify unmet clinical needs, we developed two surveys:

i) An eight-question survey was sent to each FCC representative, totalling nine representatives from 11 FCCs. The survey aimed to gain insights into how FCCs become aware of VUS reclassifications, actions taken post-notification of VUS upgrades/downgrades, and reasons for failing to update their internal clinical database.

ii) A 13-question survey was sent to each laboratory designated as being used by the FCCs, totalling six representatives from six laboratories. This survey sought to understand the reasons prompting VUS review, the frequency and constraints of VUS review, how laboratories learn of new Expert Panel classifications and subsequent actions, notifications of reclassifications to FCCs, and perspectives on the significance of regular VUS review.

Both of these surveys (Supplementary Material 1) comprised multiple-choice questions, all of which had an option to provide additional comments when considered relevant, as well as separate open-ended questions. Multiple choice responses were analysed by frequency, and the most frequent responses to open-ended responses were summarised.

## Results

### Variant spectrum per gene and ClinVar group

Data was available from 1472 patient records for breast cancer gene variants from all 11 participating FCCs, corresponding to 944 unique variants recorded as “VUS” across the FCC internal databases. Of these, the number of VUS per gene was as follows: 522 in *BRCA2*, 265 in *BRCA1*, 104 in *PALB2*, 37 in *TP53*, 11 in *PTEN*, and five in *CDH1*.

**Fig. 2:**
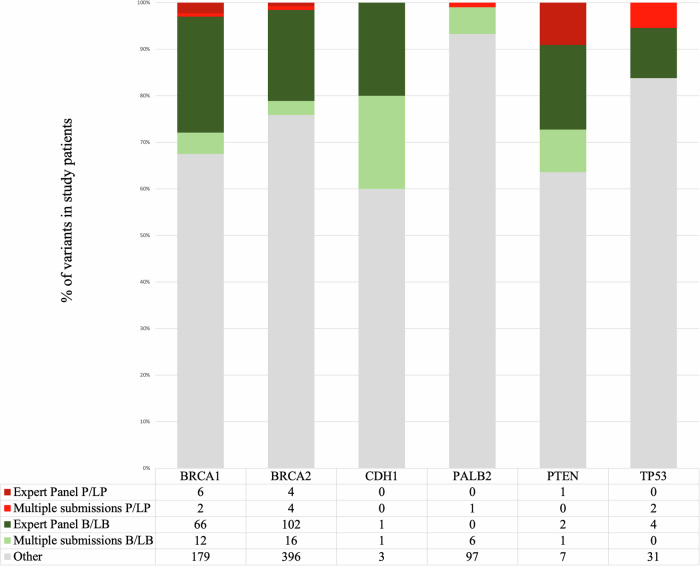
Results from the ClinVar crosscheck for 944 unique breast cancer VUS retrieved from FCCs according to the review status and designated summary classification. B/LB Benign/Likely benign, P/LP Pathogenic/Likely pathogenic.

An overview of the results from the ClinVar crosscheck is shown in Fig. 2. At the time (September 2022), approximately 20% of the unique VUS had Expert Panel submissions as P/LP [11] and B/LB (175), while 5% had multiple non-conflicting submissions in the same directions (9P/LP and 36 B/LB). For each gene, most of the VUS were in the “Other” group.

Results from the ClinVar crosscheck were returned to FCC representatives prior to the surveys, for consideration of possible reclassification review; in particular variants in ClinVar with Expert Panel assertion other than VUS, or with multiple non-conflicting submissions.

### Gene-specific ACMG/AMP application

**Fig. 3:**
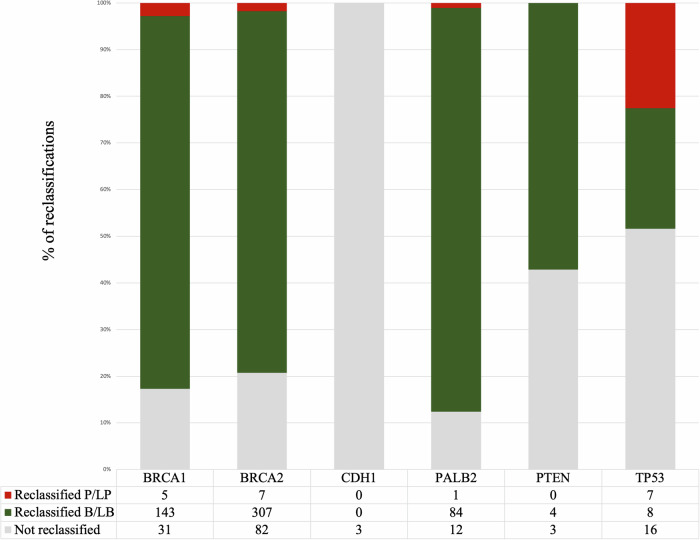
Suggested number of variant reclassifications as P/LP or B/LB out of the 713 VUS reviewed using gene-specific ACMG/AMP guidelines. B/LB Benign/Likely benign, P/LP Pathogenic/Likely pathogenic.

We applied gene-specific ACMG/AMP guidelines to all the VUS in the “Other” ClinVar group (total 713 variants). The number of our resulting proposed reclassifications per gene is shown in Fig. 3. There was no suggested reclassification for three *CDH1* variants. Application of the specifications for the remaining genes overall reclassified 3% of all variants as P/LP (20 out of the 713 “Other” VUS), and 77% variants as B/LB (546). These variants, codes applied, and our suggested classification are detailed in Supplementary Table 1. As an update, we added updated ClinVar classification details (as at 27 January 2024).

### Results from FCC survey

We received survey results from representatives of all 11 participating FCCs, with some clinicians answering on behalf of multiple centres. A simplified summary of key survey results is shown in Table 1.

**Table 1:** Summary of relevant responses from the FCC survey by nine representatives^a^.

• How FCCs report to become aware of VUS reclassifications
- Laboratory/s routinely inform them of VUS upgrades (VUS to P/LP) for reports of existing patients (4/9)
- Laboratory/s routinely inform them of VUS downgrades (VUS to B/LB) for reports of existing patients (3/9)
- There is a diagnostic report for a new patient with the same variant (3/9)
- Regular review of variants in internal clinical database (2/9)
- Other: review with the laboratory when patients contacts for more information (3), referral of a relative (3), local and/or national MDT meetings (1)
• **Clinical actions taken by FCCs after VUS reclassifications**
- VUS upgrades: Request re-issue of a report for patients with an alternative pre-existing classification (9/9)
- VUS downgrades: Request re-issue of a report for patients with an alternative pre-existing classification (6/9)
• **Reasons for not updating internal clinical database**
- Not being aware of a VUS reclassification (8/9)
- Lack of resources (3/9)
- Not clinically necessary (1/9)

*B/LB* Benign/Likely benign, *FCC* Familial cancer centre, *MDT* Multidisciplinary team, *P/LP* Pathogenic/Likely pathogenic, *VUS* Variant of uncertain significance.

^a^Responses without the denominator noted (“/”) refer to open-ended questions

Regarding how FCCs become aware of VUS reclassifications, four of the nine FCC representatives reported that their laboratory provider routinely notifies them of VUS upgrades, while this number decreased to three for downgrades. Other methods selected were receiving a diagnostic report for a new patient with the same variant (3/9) and conducting regular reviews of variants within their internal clinical database (2/9). Additionally, some FCC representatives provided free-text responses citing additional reasons such as patients reaching out for more information (3), referral of a relative (3), and involvement in local and/or national multidisciplinary team (MDT) meetings (1).

As per actions taken upon VUS reclassifications, it was found that all participating FCCs requested the re-issue of reports for patients in cases of VUS upgrades, whereas only six out of nine FCC representatives did so for variant downgrades.

Lastly, the majority of FCC representatives reported that the primary reason for the lack of updates in their internal clinical databases was their unawareness of VUS reclassifications (8/9), with a lack of resources also cited as a contributing factor by a third of the representatives (3/9), and not considered clinically necessary by one representative (1/9).

### Results from laboratory survey

We received survey results from representatives of all six participating laboratories. A summary of the responses regarding activities prompting review of previously identified VUS is presented in Table 2, with a scale provided from 1 (least often) to 5 (most often) for the activities considered relevant by the laboratories.

**Table 2:** Summary of activities most commonly prompting VUS review as reported by six laboratory representatives.

Activity	Scale from 1 (least often) to 5 (most often)						Average^a^
	Lab 1	Lab 2	Lab 3	Lab 4	Lab 5	Lab 6	
FCC/clinician contacts the lab for more/updated information	5	1	2	5	5	5	3.8
The laboratory identifies new evidence (clinical, functional etc.) for a specific variant	2	5	4	3	2	2	3.0
FCC/clinician provides information to justify variant re-review	4	2	1	1	2	4	2.3
New classification guidelines/recommendations become available	3	3	–	2	1	3	2.0
Regular VUS review as part of the laboratory SOP	5	5	–	–	–	1	1.8
A new evidence type/algorithm is included in the lab SOP	1	3	–	2	1	2	1.5
New ClinVar Expert Panel submissions are available	2	2	–	1	1	3	1.5
New ClinVar laboratory or research submissions are available	1	–	–	1	1	2	0.8
The laboratory generates or accesses external research findings	1	–	–	–	1	2	0.7

*FCC* Familial cancer centre, *SOP* Standard operating procedures, *VUS* Variant of uncertain significance.

^a^The scale of activities not considered relevant, marked with a “−”, was counted as 0 for average calculations.

The activity most commonly cited as prompting review was FCCs contacting laboratories for additional information (average rating of 3.83), followed by laboratories identifying new evidence (average rating of 3). Conversely, activities least likely to prompt VUS review included laboratories generating or accessing external research findings (average rating of 0.7), followed by the availability of new ClinVar submissions (average rating of 0.8).

A simplified summary of other key survey results from the six laboratory representatives is shown in Table 3. Only two out of six laboratories reported conducting regular reviews of VUS, one annually, the other at least biannually. Lack of resources was cited by all laboratories as a primary reason for not regularly conducting this activity. Additional reasons noted by half of the laboratories were an inability to track previous results for variants not included in reports, and the activity not deemed clinically relevant unless prompted by FCCs for additional information.

**Table 3:** Summary of other relevant responses from the diagnostic laboratory survey by six representatives^a^.

• Frequency of VUS review
- Every 1 or 2 years (2/6)
- No regular review (4/6)
• **Reasons limiting regular VUS review**
- Resources (6/6)
- Inability to track previous results for variants not included in reports (3/6)
- Not considered clinically relevant unless FCCs contact for more information (3/6)
• **How laboratories report to become aware of new Expert Panel reclassifications**
- By being members of ClinGen (2)
- No single established process (4)
• **Actions taken after laboratories become aware of a new Expert Panel reclassification**
- Review and/or discuss the information provided (3)
- Reclassify the variant (1)
- Inform the relevant FCC (1)
• **Routine notification of reclassifications to the FCCs**
- Yes for variant upgrades (6/6)
- Yes for variant downgrades (5/6)
• **Existence of a documented process in place by which VUS reclassifications are notified to the FCCs**
- Yes (5/6)
• **Prioritisation of VUS regular review if there was more funding**
- Yes (5/6)

*FCC* Familial cancer centre, *VUS* Variant of uncertain significance.

^a^Responses without the denominator noted (“/”) refer to open-ended questions.

Regarding awareness of new Expert Panel reclassifications in an open-ended survey question, two out of the six laboratories indicated that they had easier access to these updates by virtue of being Variant Curation Expert Panel (VCEP) members, while the remaining laboratories lacked a standardised process.

Another open-ended survey question revealed the following actions taken following new Expert Panel reclassifications, including: review and/or discussion of provided information for potential reclassification (3); reclassification of relevant variants (1); and informing the relevant FCC to request a reclassification from the lab (1).

With regards to notifications to FCCs, all laboratories reported notifying FCCs of VUS upgrades, compared to five out of six for downgrades. One of the laboratories reporting notifications of both options added a note to clarify that these were expected actions, since they would only review VUS following a previous FCC enquiry. The majority of laboratories reported having a documented process in place for notifying FCCs of VUS reclassifications (5/6).

Lastly, all laboratories except one indicated that VUS regular review would be prioritised if additional funding were available (5/6).

## Discussion

We have performed a nation-wide audit of VUS recorded in the clinical databases of Australian familial cancer services to determine the scale of unrecognised actionability, and to map the current and required processes to achieve effective variant review. Our ultimate aim is to inform future improvements in Australian practices concerning variant classification, reducing variant uncertainty and improving patient clinical management.

With a straightforward crosscheck of the 944 clinically detected VUS retrieved from FCCs against ClinVar, we found that a notable number of the historical reports recorded in FCC internal clinical databases had not undergone updates and/or reassessment with existing new evidence. Approximately a quarter of the so-called VUS had an Expert Panel or multiple non-conflicting submissions as P/LP or B/LB, information that was returned to the FCCs to encourage further review of these variants. For the larger group of remaining variants that are uncertain, have single or conflicting submissions in ClinVar or that are absent from the database altogether (*n* = 713), we found that application of gene-specific VCEP guidelines resulted in a 79% reduction in the VUS rate, even when relying solely on publicly available information. When combining both results, our analysis suggests that 84% of the initially provided 944 unique VUS could potentially be reclassified as P/LP or B/LB. While most of these potential reclassifications were in the benign direction (80%), there are many reasons to consider variant downgrades as clinically important, including the opportunity to avoid unnecessary clinical risk management interventions or relief from any continuing anxiety, although it can also be an indication to continue searching for alternative genetic explanations.

To better understand the factors contributing to these findings, and to describe the barriers to FCCs and laboratory identifying and implementing reclassifications, we surveyed both groups. Representatives from a large majority of FCCs (89%) indicated that they do not routinely update their internal variant database as they are generally not aware of any potential reclassifications. Additional FCC survey results indicated that 100% of VUS upgrades and 67% of downgrades, would be communicated to patients. In general, this attitude aligns with findings from a US-based survey study where both oncologists and genetic counsellors concurred that all reclassified variants, even when they do not represent a change in clinical management, should be disclosed to patients [23]. On the other hand, in the laboratory survey results, laboratories indicated that they notify the FCCs of variant reclassifications in nearly all instances, acknowledging that the primary reason prompting VUS review was an FCC request to the laboratory for additional or updated information. This highlights a major flaw in the current VUS review process: FCCs heavily rely on laboratories to keep them updated of variant reclassifications, as they do not have the clinical capacity and/or expertise to do their own reviews, but laboratories rarely initiate VUS review without a clinical prompt. The results suggest that only a relatively low proportion of VUS found in patients will be put forward for further review due to a request from the FCC or as a result of a relative undergoing testing, and that in most instances where a potential reclassification could occur based on updated information and/or classification methods, this opportunity is missed. Only two of the six surveyed laboratories indicated that they initiate their own variant re-review on a regular basis. Resources was the unanimous reason for no more regular or initiated variant re-review, and all but one of the laboratories noted that they would prioritise VUS review if they had more funding to do so. One laboratory representative elaborated that regular VUS review, regardless of the funding, requires significant consideration of the downstream clinical interactions and interventions, and highlighted concerns about possible legal implications from unrecognised actionability of VUS.

These results are a prompt to consider how a more effective and reliable process could be implemented for identifying reclassifiable VUS in clinical datasets and communicating updated curations to facilitate optimal care for patients. A simple and affordable recommendation to streamline the VUS review process between clinical services and laboratories is to promote automated notifications to the laboratories of Expert Panel reclassifications for variants within their curation system. In Australia, there is potential to facilitate this by leveraging the properties of Shariant [24], a system for real-time sharing of variant data between Australian FCCs and laboratories, which has recently introduced this capability. This could be supplemented by general look-ups to ClinVar, preferably in an automated manner as recently proposed [25]. Ideally, this approach would be coupled with encouraging proactive notification of reclassifications by laboratories to FCCs. However, Expert Panels do not typically perform large-scale reviews of variants, as this typically exceeds the capacity of the VCEPs and is not prioritised by ClinGen, which usually focuses on variants with multiple uncertain or conflicting submissions. Therefore, while this “ClinVar notification” approach can be beneficial, it will not address the larger number of variants that are not reviewed by Expert Panels but would still benefit from more frequent laboratory-based reviews following gene-specific ACMG/AMP guidelines.

There was no clear consensus in the survey responses around which group bears the final responsibility for the task of continuing review of VUS to identify potential reclassifications. A more comprehensive solution is required for promoting periodic laboratory review of VUS for potential increased actionability. We expect that the reclassification rate of this study would be greater with inclusion of evidence held privately within laboratories. The reclassification rate is also likely to increase with evolution of ClinGen gene-specific classification guidelines, which has been demonstrated in several VCEP-related studies to reduce the VUS rate [26–28].

Regular VUS review and variant reclassification will require resources, adding to the routine work of trained staff. Numerous studies have demonstrated the cost-effectiveness of genetic testing in women with breast cancer [29–31]. In response, countries such as Australia and the UK have integrated genetic testing for hereditary breast cancer risk into their respective government funded health services (Medicare and the NHS), targeting patients meeting specific eligibility criteria, including those with a 10% risk of carrying a germline pathogenic variant based on clinical presentation. It should be noted that VUS review and variant reclassification activities to identify a P/LP variant should be less costly than the initial genetic testing that incurred the costs of the initial consultation, sample collection, laboratory analysis, sequencing bioinformatics. Costs associated with VUS review would be limited to resources for re-curation costs, and re-issue of reports when necessary. Additionally, reclassifying VUS as B/LB (80% reclassification rate as per our work) has the potential to lead to savings by avoiding the cost of unnecessary clinical interventions, recognising that not all VUS are considered clinically relevant and management might be dependent on other factors, and by removing variants from the list of VUS requiring ongoing review by the laboratory. Further, for VUS previously returned to patients, there is potential to provide reassurance and relieve anxiety following a B/LB reclassification. Therefore, publicly funding the VUS review process in countries where hereditary cancer testing is already publicly funded appears likely to have economic benefits. In terms of VUS review frequency, two of the laboratories reported conducting this activity every 1-2 years, aligning with reported most effective frequencies [32, 33]. The inconsistency in approaches between diagnostic laboratories reveals a need to develop a national approach to standardise variant reclassification protocols. The consensus framework on variant reclassification developed by CanVIG-UK [34] might provide a useful starting point in this regard.

Future directions of this work include assessing the clinical impact of our findings, and the cost-effectiveness of implementing routine variant review and reclassification practices. In parallel, national efforts should be directed towards promoting collaboration between FCCs and laboratories to ensure timely notification processes, potentially through standardised protocols and increased communication channels.

## Supplementary information

Survey sent to Familial Cancer Centres (a) and Survey sent to Laboratories (b)

Suggested clinically-relevant classifications from application of gene-specific ACMG/AMP criteria and points system (a-e)

## Data Availability

Numerous data are made available via supplementary materials. Additional data can be made available on reasonable request.
