# Supplementary material for: Unrecognised actionability for breast cancer risk variants identified in a national-level review of Australian familial cancer centres
Source: Eur J Hum Genet. 2024 Oct 14;32(12):1632–9. doi: 10.1038/s41431-024-01705-9 (PMC11607406; doi:10.1038/s41431-024-01705-9)
Supplement: Supplementary file 1 — Survey sent to Familial Cancer Centres (a) and Survey sent to Laboratories (b) [file 41431_2024_1705_MOESM1_ESM.pdf]

Supplementary Material 1a. Survey sent to Familial Cancer Centres

Practices around VUS re-evaluation and re-reporting

Background

This survey presents questions to assess current practices and policies of representatives from **Australian clinical genetics services** relating to re-evaluation of variants originally identified as Variants of Uncertain Significance (VUS), and stored in patient clinical databases. The survey is comprised of 8 questions, and should take less than 15 minutes to complete, by Survey Monkey or Zoom interview with survey co-ordinators, as preferred.

Results will be used to inform interpretation of findings from a parallel survey assessing practices of selected Australian clinical testing laboratories around re-evaluation and re-reporting of variants originally reported as VUS, initiated to explore findings from an NBCF-funded project centred on classification of germline variants in hereditary breast cancer genes. The aim of the combined surveys is to identify areas of unmet need in the laboratory and clinical setting, and thereby help direct future resource development and funding for VUS re-evaluation.

Individual responses will be reviewed and collated by survey co-ordinators Cristina Fortuno (Cristina.Fortuno@qimrberghofer.edu.au; Amanda Spurdle (Amanda.Spurdle@qimrberghofer.edu.au). Please note that information that could identify you such as your name, contact details, etc. will be replaced by a random code to protect your identity (a process known as de-identification) for your survey response. Please note that data collected will be retained at QIMR Berghofer indefinitely to comply with relevant laws and legislations, including the Public Records Act 2002. De-identified summary findings will be returned to the survey participants, and discussed with study collaborators as relevant to the purpose of this survey - to identify areas of unmet clinical need in Australia. Summary findings may be included in a publication and this information will not be linked in any way to your survey responses.

Your participation in this survey is entirely voluntary and you can choose not to participate. If you do choose to participate, you can withdraw from the study at any time. However, if you withdraw your consent after survey completion, data already collected will be retained to comply with laws as stated above, but you may request that your data be excluded from analysis. You can withdraw your consent by contacting the survey co-ordinators. Please be aware that by providing responses to this survey, you will consent to the use of the survey summary results, as described above.

The conduct of this survey is covered under QIMR HREC Approval P1051. If you have any concerns or complaints regarding the conduct of this study, you may contact the Chairperson of the QIMR Berghofer Medical Research Institute Human Research Ethics Committee (QIMRB HREC) via the Committee Secretary on Tel: 07-3362-0117.

Survey Questions

1. **Please provide your name, and the name of the clinical service relevant to this survey response**

2. **How do you become aware of updates to the clinical classification of gene variants in your clinical database? Check all that apply**
    - Laboratories routinely inform you of any VUS upgraded classifications (VUS to L/P) for reports relevant to existing patients
    - Laboratories routinely inform you of any VUS downgraded classifications (VUS to L/B) for reports relevant to existing patients
    - Laboratories routinely inform you of L/P downgraded classifications (L/P to VUS or L/B) for reports relevant to existing patients
    - There is a new report (new patient) for an existing variant in your database

- Regular review
- Other (please specify including other comments and considerations)

3. **What of the following actions do you regularly take after you become aware of a change in a variant classification from VUS to L/P? Check all that apply**
    - Request re-issue of a report for patients with an alternative pre-existing classification
    - Reflect this update for all relevant patients in your clinical database
    - Notify patients with an alternative pre-existing classification
    - Other (please specify other comments and considerations around factors that influence your decisions to take any of the actions above)

4. **What of the following actions do you regularly take after you become aware of a change in a variant classification from VUS to L/B? Check all that apply**
    - Request re-issue of a report for patients with an alternative pre-existing classification
    - Reflect this update for all relevant patients in your clinical database
    - Notify patients with an alternative pre-existing classification
    - Other (please specify other comments and considerations around factors that influence your decisions to take any of the actions above)

5. **In cases where you don't update your clinical database with new classifications, what are the main contributing factors? Check all that apply**
    - Lack of resources (staff, money, etc.)
    - Not clinically necessary
    - You were not aware of any change in classification
    - Other (please specify other comments and considerations around factors that influence your decisions take any of the actions above)

6. **Referring to the Excel file we shared, please provide answers for each of these points in the answer box below, for all the variants we highlighted as having a L/P classification in ClinVar from your clinical database:**
    - How many represent newly identified changes in classifications that you were not previously aware of?
    - How many patient entries were consequently updated in your clinical database?
    - Please include other comments and considerations around factors that influence your decisions take any of the actions above

7. **Referring to the Excel file we shared, please provide answers for each of these points in the answer box below, for all the variants we highlighted as having a L/B classification in ClinVar from your clinical database:**
    - How many represent newly identified changes in classifications that you were not previously aware of?
    - How many patient entries were consequently updated in your clinical database?
    - Please include other comments and considerations around factors that influence your decisions take any of the actions above

8. **Any other comments and considerations regarding factors that would assist you in maintaining currency in classification for variants in your clinical database**

Supplementary Material 1b. Survey sent to Laboratories

Practices around VUS re-evaluation and re-reporting

Background

This survey presents questions to assess current practices and policies of **Australian clinical testing laboratories** relating to re-evaluation and re-reporting of variants originally identified as Variants of Uncertain Significance (VUS) by clinical genetic testing. The survey is comprised of 12 questions, and should take less than 30 minutes to complete, preferably by Zoom interview with survey co-ordinators.

Results will be used to inform interpretation of findings from a parallel survey assessing practices of selected Australian clinical genetics services around re-evaluation of VUS in patient clinical databases, initiated to explore findings from an NBCF-funded project centred on classification of germline variants in hereditary breast cancer genes. The aim of the combined surveys is to identify areas of unmet need in the laboratory and clinical setting, and thereby help direct future resource development and funding for VUS re-evaluation.

Individual responses will be reviewed and collated by survey co-ordinators Cristina Fortuno (Cristina.Fortuno@qimrberghofer.edu.au; Amanda Spurdle (Amanda.Spurdle@qimrberghofer.edu.au). Please note that information that could identify you such as your name, contact details, etc. will be replaced by a random code to protect your identity (a process known as de-identification) for your survey response. Please note that data collected will be retained at QIMR Berghofer indefinitely to comply with relevant laws and legislations, including the Public Records Act 2002. De-identified summary findings will be returned to the survey participants, and discussed with study collaborators as relevant to the purpose of this survey - to identify areas of unmet clinical need in Australia. Summary findings may be included in a publication and this information will not be linked in any way to your survey responses.

Your participation in this survey is entirely voluntary and you can choose not to participate. If you do choose to participate, you can withdraw from the study at any time. However, if you withdraw your consent after survey completion, data already collected will be retained to comply with laws as stated above, but you may request that your data be excluded from analysis. You can withdraw your consent by contacting the survey co-ordinators. Please be aware that by providing responses to this survey, you will consent to the use of the survey summary results, as described above.

The conduct of this survey is covered under QIMR HREC Approval P1051. If you have any concerns or complaints regarding the conduct of this study, you may contact the Chairperson of the QIMR Berghofer Medical Research Institute Human Research Ethics Committee (QIMRB HREC) via the Committee Secretary on Tel: 07-3362-0117.

Survey Questions

1. **Please provide your contact name, email address, and laboratory**

2. **Do you have a tracking system in place to keep records of VUS identified, including the corresponding clinical service/clinician requesting the test, and patient identification number?**
   - Yes
     - o all variants prioritised for curation
     - o only fully curated variants
     - o only reported variants
   - If Yes, are these all in a single data system?
   - No
   - Comments

3. **What prompts re-review of previously identified VUS? Check all that apply, and note on a scale of 1 (not often) to 5 (very often)**
   - Clinical service/clinician contacts the laboratory for more/updated information
   - Clinical service/clinician provides information to justify variant re-review
   - The laboratory identifies new evidence (clinical, functional etc.) for a specific variant
   - The laboratory generates or accesses external research findings
   - A new evidence type/algorithm is included in the laboratory SOP
   - New classification guidelines/recommendations become available
   - New ClinVar Expert Panel submissions are available
   - New ClinVar laboratory or research submissions are available
   - Regular VUS re-review as part of the laboratory SOP
   - Other – please describe

4. **How do you become aware of a new ClinGen Variant Curation Expert Panel reclassification for a previously identified VUS?**

5. **What actions do you take when you become aware of a new ClinGen Variant Curation Expert Panel reclassification for a previously identified VUS?**

6. **If regular re-review is part of your laboratory SOP, please state frequency**

7. **What limits regular re-review of previously identified VUS? Check all that apply, and note on a scale of 1 (not important) to 5 (very important)**
   - Not considered clinically relevant unless clinical services/clinicians contact laboratory for (or with) information
   - Inability to track previous results for variants not included in reports
   - Lack of policies and guidelines
   - Resources
   - Other – please explain

8. **Do you have a documented process in place by which VUS reclassifications in your laboratory are notified to the clinical service/referring clinician whose patient had a VUS had been previously identified**
   - Yes
   - No
   - Other – please explain

9. **Do you always notify clinics/clinicians about a reclassification to L/P for a VUS previously identified for a patient?**

   9.1 If yes, do you do for just one patient or all patients with that VUS in your database?

   9.2 Is a report always re-issued? If not, why not?

   9.3 If not always for all relevant patients or not consistently, why not?

10. **Do you always notify clinics/clinicians about a reclassification to L/B for a VUS previously identified for a patient?**

    10.1 If yes, do you do for just one patient or all patients with that VUS in your database. If not for all relevant patients, why not?

    10.2 Is a report always re-issued? If not, why not?

10.3 If not always for all relevant patients or not consistently, why not?

11. If you re-issue a report, who pays for the report, and what is the cost?

12. Would you prioritise VUS regular review if you had more funding?

- Yes
- No
- Please provide additional comments that you might consider relevant

12. 1. If relevant, what information / metrics would be helpful to justify increased funding to your laboratory to cover (routine) VUS re-evaluation and re-reporting?
